# Assessment of disease severity in hospitalized community-acquired pneumonia by the use of validated scoring systems

**DOI:** 10.1186/s12890-025-03550-y

**Published:** 2025-03-03

**Authors:** Sandleen Iftikhar, Bjørn Waagsbø

**Affiliations:** 1 Department of Pulmonary Disease, St. Olavs University Hospital, Trondheim, Norway; 2 Regional Competence Centre for Hygiene, Regional Health Trust Mid, Trondhjem, Norway; 3 Antimicrobial Stewardship Team St. Olavs University Hospital, Trondheim, Norway

**Keywords:** Community-acquired pneumonia, Severity assessment, Antimicrobial stewardship, Antimicrobial therapy

## Abstract

**Background:**

Severity assessment of community-acquired pneumonia (CAP) is essential for many purposes. Among these are the microbiological confirmation strategy and choice of empirical antimicrobial therapy. However, many severity assessment systems have been developed to aid clinicians to reach reliable predictions of severe outcomes.

**Methods:**

We aimed to apply nine disease severity assessment scoring systems to a large 2016 to 2021 CAP cohort in order to achieve test sensitivity, specificity and predictive values. We used intra-hospital all-cause mortality and the need for intensive care admission as outcomes. The area under the receiver operating characteristic (ROC) curve was used to display test performance.

**Results:**

A total of 1.112 CAP episodes were included in the analysis, of which 91.4% were radiologically, and 43.7% were microbiologically confirmed. When intra-hospital all-cause mortality was set as outcome, tests designed for CAP severity assessment, like PSI, and CURB65 outperformed the more generic systems like NEWS2, qSOFA, SIRS and CRB65. Designated tests for CAP (PSI, IDSA/ATS and CURB65) and overall critical illness (SOFA) displayed acceptable performances as compared to non-specific tests. Comparable results were gained when intensive care admission was set as outcome. The area under the receiving operating curve was 0.948, 0.879, 0.855 and 0.726 for the SOFA, PSI, IDSA/ATS and CURB65 scoring systems, respectively.

**Conclusion:**

CAP severity assessment remains important. Designated CAP severity assessment tools outperformed generic tests.

**Supplementary Information:**

The online version contains supplementary material available at 10.1186/s12890-025-03550-y.

## Introduction

Community acquired pneumonia (CAP), albeit a common infection, can be a potential life-threatening illness and is the most common causes of sepsis [1, 2]. It is associated with high morbidity and mortality rates especially in the elderly and in patients with underlying comorbidities [3].

Hospital admission rates can vary widely and are often not directly related to disease severity. A number of factors contribute to decisions on site of care and level of therapy, among these medication compliance, ability to maintain oral intake, cognitive or functional impairment, social circumstances, disease severity, and organ support measures. Clinicians can misinterpret or misjudge disease severity, leading to unwarranted therapy for relatively mild cases, or missed or delayed therapy for more severe cases. However, the risk of short-term mortality in CAP is more likely to be over-, rather than underestimated, when using simplified disease severity assessment scores [4].

The initiation of empirical antimicrobial therapy and the site of care are by most professional guideline recommendations determined by CAP severity at presentation. However, the evidence to support a standardized approach with the use of disease severity assessments for CAP, is still sparse in terms of improved outcomes [5]. In countries with low rates of antimicrobial resistance, unwarranted broad-spectrum antimicrobial therapy is of particular concern.

A wide array of supporting systems have been developed and validated to aid clinicians when assessing disease severity in especially CAP. Of these systems, some are relatively effortless, while some are complex. In this study, we have applied these systems to a CAP-cohort, and established test properties and performance.

## Patients and methods

### Study setting

A single-center, 1.000 bed, university teaching hospital in mid-Norway, accepting all patient categories, except transplantation surgery.

### Study population

We identified all cases of CAP admitted to a university teaching hospital in Norway between 2016 and 2021. Due to labor-intensive registrations, only the months between March and May and the departments of medicine and pulmonology were eligible for inclusion. Months were chosen to represent an influenza-diminished period, regular hospital staffing situations, and standardized laboratory services.

Final discharge diagnoses (ICD-10 between J13 to J18.9) were used to identify eligible cases for inclusion. We have earlier reported patient characteristics, aetiology, resistance patterns and antimicrobial therapy to these case series [6].

### Study outcomes

The primary outcome of this particular study was to report the performance of established, and commonly used, clinical scoring systems for disease severity assessment in the emergency room setting for CAP patients. Sensitivity, specificity, and predictive values were calculated using intra-hospital all-cause mortality and ICU-admission as outcomes. Area under the receiver operating curve (AUROC) were used to depict performance of the assessments strategies for intra-hospital all-cause mortality.

### Data collection

All data registered were collected retrospectively after each ensuing year between 2016 and 2021. Included variables were patient characteristics, clinical characteristics present at admission, radiological and laboratory findings, antimicrobial therapy, and clinical outcomes.

### Severity assessments

In Table 1 we have presented the clinical scoring systems that were selected by the study group. We have provided all subcriteria used in the appendix.

**Table 1:** Selected scoring systems for disease severity

System^a^	Year launched	Subcriteria^b^	Reference^c^	Validation^d^
qSOFA	2016	3	[7]	[8–11]
CRB65	-	4	-	[12–14]
CURB65	2003	5	[5]	[15, 16]
SIRS (Sepsis 1)	1992	4	[17]	[18, 19]
NEWS2	2017	7	[20]	[21–23]
SOFA (Sepsis-3)	2016	6	[24]	[25–27]
PSI	1997	20	[14]	[15, 28]
IDSA/ATS	2007	11	[29]	[30–33]
Sepsis-2	2003	A myriade	[34]	-

^a^See appendix for full outlining of system name and subcriteria

^b^Number of subcriteria included in system

^c^Reference to the original publication of the system

^d^Relevant validation studies

When assessing disease severity by the use of qSOFA, CRB65, CURB-65, SIRS, and NEWS2 we calculated sensitivity, specificity, and predictive values by extracting the necessary subcriteria directly from the collected data. To the best of our knowledge and experience, we appointed these five scoring systems as frequently used in clinical practice. The four remaining scoring systems, at the bottom of Table 1, were appointed infrequently used.

When assessing consciousness, we considered new-onset confusion, disorientation, agitation, responds to voice, responds to pain, or unresponsive as relevant. To some extent, we used clinical judgement to deem the level of affected consciousness.

When calculating the initial SOFA-score, we frequently used the arterial partial pressure of oxygen (P_a_O_2_) instead of P_a_O_2_/F_i_O_2_. In cases initially lacking measurements of P_a_O_2_, we imputed peripheral saturation of oxygen (SO_2_) to the calculation. This has earlier been demonstrated to accurately correlate and provide acceptable outcomes [35]. We were able to calculate the SOFA-score to 96.4% of included cases.

The PSI is much more detailed as 20 subcriteria are needed to calculate the score. A great proportion of these subcriteria are related to comorbidity status, to which we used some extent of clinical judgement. In particular, we deemed prior or present comorbidities, the stage and severity of comorbid illness, and the effect of instituted therapy. This represents, in deed, everyday clinical practice. None of the cases included were nursing home residents. We also used the serum creatinine level at > 120 µmol/L to represent new-onset kidney dysfunction instead of blood urea nitrogen concentration. To assess the haematocrit value we imputed three-folded haemoglobin levels according to earlier practice [36]. PSI-score was ultimately calculated to 90.7% of included cases.

The 2007 IDSA/ATS clinical practice guideline for CAP stated a set of major or minor criteria for the disease severity assessment. The fulfilment of one major or at least three minor criteria would tentatively imply severe CAP. The minor criteria resemble CURB65-criteria, and the major criteria are invasive mechanical ventilation or septic shock with the need for vasopressors. An initial score could be established to 94.5% of included cases.

We also set out to include the 2001 international sepsis definition and case criteria (Sepsis-2) in this study. The case criteria are aggregated from multiple variables, including general, inflammatory, hemodynamic, organ dysfunction, and tissue perfusion variables. In contrast to others systems, there is no established or suggestive level of number of subcriteria to fulfil the case criteria. Instead, judicious and extensive clinical judgement from the bedside attending doctor, to evaluate the myriad of presenting signs and symptoms, determines whether the infection is severe or not. Because this extensive individual evaluation universally was poorly documented in our study, we were unable to calculate the score for all inclusions.

### Statistical analyses

To calculate sensitivity, specificity, and predictive values we used cross tabulation functions in IBM SPSS (Statistical Package for the Social Sciences), version 29. We defined thresholds for positive or negative test, which are summarized in Table 2.

**Table 2:** Thresholds for positive test

System	Test threshold for positive test
qSOFA	2 or more subcriteria
CRB65	2 or more subcriteria
CURB65	3 or more subcriteria
SIRS (Sepsis 1)	2 or more subcriteria
NEWS2	5 or more points
SOFA (Sepsis-3)	Increase of 2 or more points
PSI	Risk class II with 90 or more points
IDSA/ATS	One major or 3 or more minor criteria
Sepsis-2	No threshold established

The performance of the scoring systems were calculated by the use of area under the receiver operating curve (AUROC).

### Ethical considerations

The study group has previously been granted approval by the hospital administration and data protections officials to conduct studies on lower respiratory tract infections. We also received approval by the Regional Committee for Medical and Health Research Ethics (REK 2017/1439), stating that inclusion consent was deemed unnecessary due to retrospective study design.

## Results

### Patient characteristics and outcomes

Over six years we included 1.112 patients in this study. All patients were ultimately diagnosed and discharged from hospital with CAP as a primary diagnosis, of which 91.4% were radiologically, and 43.7% microbiologically confirmed, on average for all years. We have previously reported patient characteristics, aetiology, resistance patterns and antimicrobial therapy in this case series [6]. Among included cases, mean age was 70.3 years and nearly 40% were aged above 65 years. Table 3 summarizes relevant characteristics and outcomes.

**Table 3:** A selection of patient characteristics and outcomes of studied inclusions

Characteristics and outcomes		
Age	Mean	70.3 years
	Proportion > 65 years	67.5%
Gender	Male	45.5%
Comorbidities	Median number of conditions	3
	Median Charlson comorbidity index	4
ICU	Proportion admitted	6.1%
	Invasive ventilation	4.7%
Sepsis^1^	Without shock	9.9%
	With shock	1.9%
Length of stay	Median and interquartile range	7 (5–9)
All-cause mortality	In-hospital	10.9%
	30-day	14.3%
	90-day	23.8%

^1^According to the 2016 international consensus definitions (Sepsis-3)

### Intra-hospital all-cause mortality

Firstly, we calculated sensitivity, specificity and predictive values to a positive test when the outcome was intra-hospital all-cause mortality. Table 4 summarizes the calculations.

**Table 4:** Results when outcome is intra-hospital all-cause mortality

Test system	*n*	Data	Sensitivity	Specificity	PPV	NPV
qSOFA	1112	100%	14/117 (12.0%)	911/995 (91.6%)	14.3%	89.8%
CRB65	1112	100%	36/117 (30.8%)	737/995 (74.1%)	12.2%	90.1%
SIRS (Sepsis 1)	1112	100%	84/117 (71.8%)	321/995 (32.3%)	11.1%	90.7%
NEWS2	1112	100%	89/117 (76.1%)	377/995 (37.9%)	12.6%	93.7%
CURB65	1112	100%	55/117 (47.0%)	957/995 (96.2%)	59.1%	93.9%
SOFA (Sepsis-3)	1072	96,4%	90/96 (93.8%)	916/976 (93.9%)	60.0%	99.3%
PSI	1009	90,7%	76/88 (88.6%)	847/921 (92.0%)	47.8%	98.8%
IDSA/ATS	1112	100%	88/101 (87.1%)	961/1011 (95.1%)	63.8%	98.7%
Sepsis-2	0	0%	NA*	NA*	NA*	NA*

*Not applicable

Sensitivity was low and specificity was somewhat reciprocally high among frequently used generic tests, like qSOFA, CURB65, CRB65, SIRS and NEWS2, to assess disease severity when in-hospital all-cause mortality was the outcome. Among the more infrequently used tests in Norway that require more extensive data entries, like SOFA, PSI or IDSA, sensitivity and specificity were considerable higher, all reaching > 87%. The predicted positive values were low for most tests, whilst the negative predicted values were all > 89%. Calculations in accordance with the Sepsis-2-criteria were universally unattainable.

### Intensive care admission

Secondly, we calculated sensitivity, specificity and predictive values to a positive test when the outcome was ICU-admission from CAP. Table 5 summarizes the calculations.

**Table 5:** Results when the outcome is need for intensive care admission

Test system	*n*	Data	Sensitivity	Specificity	PPV	NPV
qSOFA	1112	100%	20/68 (29.4%)	966/1014 (92.5%)	20.4%	95.3%
CRB65	1112	100%	44/68 (64.7%)	794/1044 (76.1%)	15.0%	97.1%
SIRS (Sepsis 1)	1112	100%	58/68 (85.3%)	344/1044 (33.0%)	7.7%	97.2%
NEWS2	1112	100%	55/68 (80.9%)	392/1044 (37.5%)	7.8%	96.8%
CURB65	1112	100%	33/91 (36.1%)	961/1021 (94.1%)	35.5%	94.3%
SOFA (Sepsis 3)	1072	96,4%	45/68 (66.2%)	484/1044 (46.4%)	7.4%	95.5%
PSI	1009	90,7%	70/78 (89.7%)	882/931 (94.7%)	58.8%	99.1%
IDSA/ATS	1112	100%	81/88 (92.0%)	881/963 (91.5%)	49.7%	99.2%
Sepsis-2	0	0	NA*	NA*	NA*	NA*

*Not applicable

Sensitivity and specificity varied considerably among frequently used tests to assess disease severity when the need for ICU admission was the outcome. Among the more infrequently used tests that require more extensive data entries, sensitivity and specificity also varied, albeit all reaching > 66%. The predicted positive values were universally low, whilst the negative predicted values were all > 95%.

### Area under the receiver operating curve

We estimated the area under the receiver operating characteristic (AUROC) curve for the calculations when the outcome was intra-hospital all-cause mortality. The frequently used severity assessment scoring systems, like qSOFA, CRB65, SIRS and NEWS2, performed poorly as compared to CURB65, SOFA and IDSA/ATS. The AUROC-curves are shown in Table 1. The estimated area for these curves all achieved values above 0.73, which were statistically significant. The area results are provided in Table 6. The AUROC when ICU-admission was set as outcome is provided in the appendix Figure 1.

**Fig. 1:**
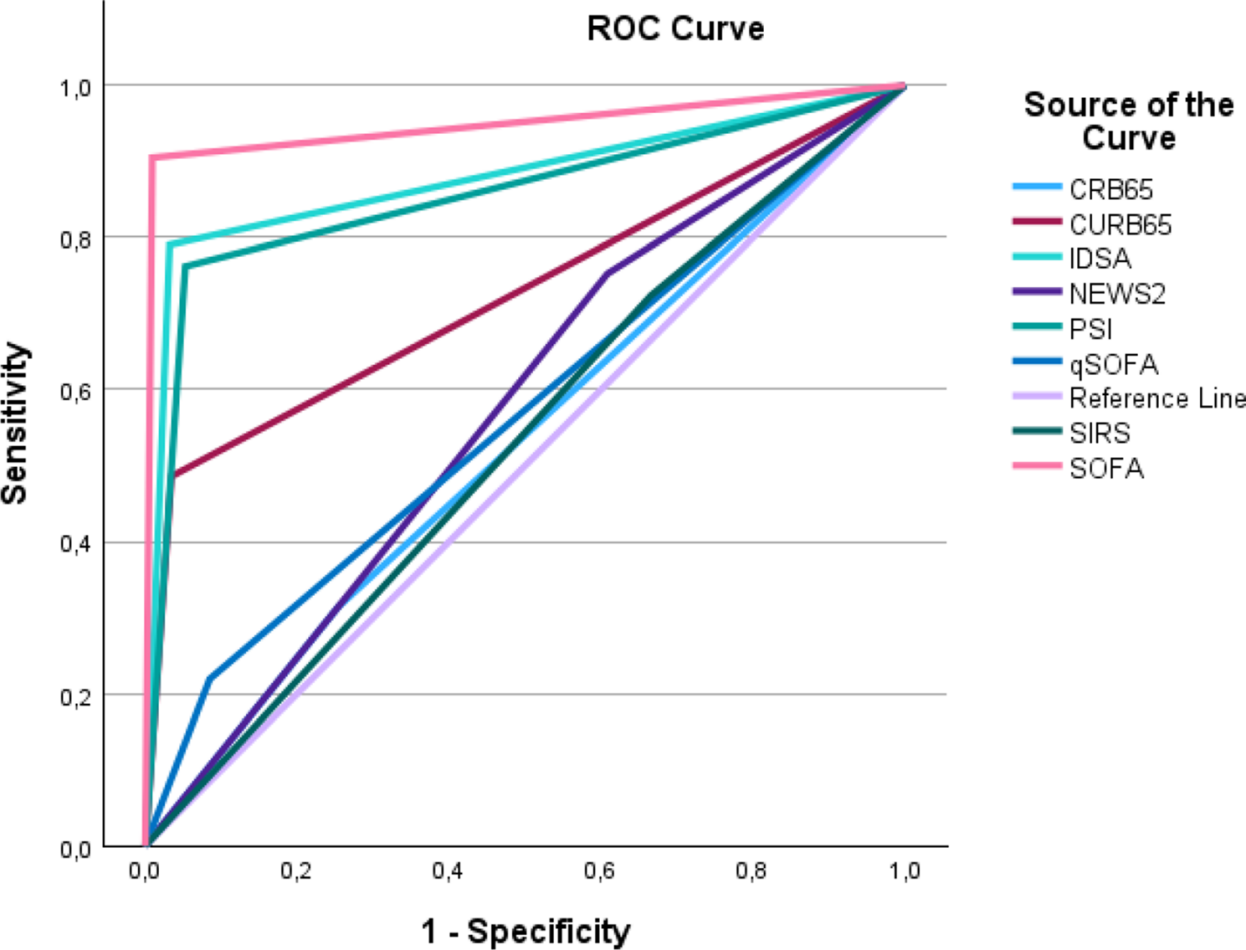
AUROC-curves for all scores are shown

**Table 6:** AUROC table for intra-hospital all-cause mortality

Test system	Area	Std error	*p*	95% CI
SOFA	0.948	0.017	0.0001	0.92–0.98
IDSA/ATS	0.879	0.024	0.0001	0.83–0.93
PSI	0.855	0.025	0.0001	0.81–0.90
CURB65	0.726	0.032	0.0001	0.66–0.79
NEWS2	0.572	0.028	0.011	0.52–0.63
qSOFA	0.567	0.032	0.34	0.51–0.63
CRB65	0.530	0.030	0.32	0.47–0.59
SIRS	0.529	0.029	0.32	0.47–0.59

## Discussion

In this study, we have demonstrated the low specificity and positive predicted value of frequently used strategies, like qSOFA, CURB65, CRB65, SIRS and NEWS2, to assess disease severity in CAP. On the other hand, more sophisticated and complex systems like the SOFA-, PSI- or IDSA/ATS-criteria, provide superior results in terms of both sensitivity, specificity and predicted values. The results pinpoint limitations of simplified disease severity scoring systems, and underscore the importance of judicious clinical assessment by the skilled clinician.

Validation studies of clinical scorings systems in the infection severity assessment have shown various results. They have also been applied to various patient populations, at various location settings, and to predict various outcomes. We used intra-hospital all-cause mortality and ICU-admission as outcomes, and concluded that qSOFA, CRB65, SIRS and NEWS2 all provided inferior AUROC (~ 0.50), and CURB65, SOFA, PSI and IDSA/ATS superior AUROC (> 0.73).

Compared to validation studies of the CURB65, our data provided less powerful sensitivity, predicted values and AUROC [5, 15, 16]. Our study design differ on some important areas, which can imply selection bias. We included no nursing home residents, and we chose to include patients admitted only from March to May for each year. Proportions that needed ICU admission, and mechanical or invasive ventilation were considerably lower than stated in the literature [12]. Of importance, disease severity assessment by the use of scoring systems were uniformly calculated by the study group retrospectively. We learned that disease severity very rarely was systematically documented, and this might imply further bias.

Disease severity assessment is crucial for many reasons. Firstly, the initiation of empirical antimicrobial therapy is often based on the assessment of disease severity, as is also the choice of antimicrobial regimen [37]. Secondly, recommendations on the timing of antimicrobial therapy administration vary according to disease severity [38]. Thirdly, strategy to establish reliable microbiological aetiology is linked to disease severity assessment in infections [39]. Fourthly, disease severity assessment determines site of care, both for community- or hospital settings. And fifthly, diseases severity assessments are prerequisites for determining overall therapy duration, oral transition, advanced diagnostic and escalated therapeutic approaches, hospital discharge, and more.

Clinical scoring systems to assess disease severity are attempts to provide the attending clinician with information to judge infections especially in the emergency room setting. Of importance, most systems was originally derived in patients already suspected of having infection [40]. The CURB65-, CRB65-, PSI- and the IDSA-criteria targeted lower respiratory tract infections specifically, while all other systems aimed to be applicable regardless of infection site. However, all systems tend to simplify complex processes of infection, inflammation and pathophysiology of heterogeneous patient groups, pathogens, and infection sites [41]. Of importance, other circumstances also affect outcomes, among these are time to diagnosis, time to antimicrobial therapy [40] and prevalence of antimicrobial resistance [42].

Importantly, the various clinical scoring systems have been developed and validated with much of the same discrete subcriteria, but at very different levels for positivity. A typical example is the respiratory rate criterion that has a level for positivity that vary by almost 40% between scoring systems [7]. Also, the more complex scoring systems, that require more data entries, are developed in conjunction with clinical judgement [29]. A frequently cited meta-analysis of the IDSA/ATS-criteria reporting one major or three minor criteria had a pooled sensitivity of 84% and a specificity of 78% for predicting ICU admission [43]. On the other hand, without a major criterion, a threshold of three or more minor criteria had a pooled sensitivity of 56% and specificity of 91% for predicting ICU admission [44].

The Sepsis-2-criteria were generally appraised by physicians when launched in 2003 [34]. According to this, the skilled physician should judiciously and comprehensively evaluate the myriad of signs and symptoms of possible sepsis to establish a reliable sepsis-diagnosis. Arbitrary criteria were thereby abandoned, and physician autonomy was re-established and accentuated. On the other hand, the Sepsis-2-criteria were challenging to operationalize into a decision support tool for less skilled clinicians. Since there is no threshold for the number of criteria fulfilled in Sepsis-2, we were unable to calculate test specifics and performance for our cohort.

Oversimplification has been the mainstay of criticism to disease severity assessment systems for CAP in particular, and infections in general [45]. Simplified systems for complex pathophysiological events may fail to correctly address the involvement or escalation of organ dysfunction, especially respiratory failure.

Our study has several important limitations. The collection of data by retrospective methodology for disease severity assessment might be inaccurate, because of temporal clinical changes, sometimes over short time periods. To some extent the actual values needed to calculate the representative score, depended on the attending medical doctors ability to document this. Moreover, the more complex scorings systems like SOFA and IDSA/ATS, have subcriteria that normally requires intensive care settings to initiate, like invasive ventilation and circulatory shock therapy. They are therefore more likely to predict severe outcomes rather than physiological or inflammatory subcriteria. The studied cohort needs to be viewed with special considerations, and results are not hurriedly generalizable. Of particular note, we did not include management or treatment details for the cohort. Cases with a definite viral aetiology were not included in the identification criteria. ICD-10 coding was used to identify eligible cases, and the final diagnosis was determined by attending physicians at the ward. Of note, a previous study has shown that 15.8% pneumonia-coded cases in fact were likely to have other diagnoses [46]. In addition, ICU-admission may not be an appropriate disease severity marker for all CAP patients, especially in the setting with advanced age and comorbidity.

## Conclusions

In conclusion, we have here demonstrated that scoring systems specifically designed for CAP severity assessment, like PSI and CURB65, outperformed the more generic systems like NEWS2, qSOFA, SIRS and CRB65. In addition, AUROC performance in prediction of in-hospital all-cause mortality, was highest for the three designated CAP-scores (PSI, IDSA/ATS and CURB65) and one critical illness score (SOFA). It is our belief that clinicians should assess CAP severity judiciously and comprehensively by the use of severity assessment scoring systems in conjunction with clinical judgement.

## Electronic supplementary material

Below is the link to the electronic supplementary material.

Supplementary Material 1

## Data Availability

The datasets used and analyzed during the current study are available from the corresponding author on reasonable request.

## Abbreviations

CAP: Community-acquired pneumonia
ROC: Receiver operating curve
AUROC: Area under the receiver operating curve
ICD-10: International classification of disease 10
qSOFA: Quick-SOFA or the quick sequential organ failure assessment
CRB65: Confusion, respiratory rate, blood pressure, and age above 65 years
CURB65: Confusion, blood urea, respiratory rate, blood pressure, and age above 65 years
SIRS: Systemic inflammatory response system
NEWS2: National early warning score 2
SOFA: Sequential organ failure assessment score
PSI: Pneumonia severity index
IDSA/ATS: Infectious diseases society of America and the American thoracic society
Sepsis-2: Sepsis definition by the SCCM (Society of critical care medicine), ESICM (European Society of intensive care medicine), ACCP (American college of chest physicians), ATS (American thoracic society), SIS (Surgical infection society)
