# Supplementary material for: Assessment of disease severity in hospitalized community-acquired pneumonia by the use of validated scoring systems
Source: BMC Pulm Med. 2025 Mar 3;25:100. doi: 10.1186/s12890-025-03550-y (PMC11877700; doi:10.1186/s12890-025-03550-y)

Appendix

# Area under the operating receiver curve

*Receiver operating curve for all scorings systems when intensive care admission was set as outcome*


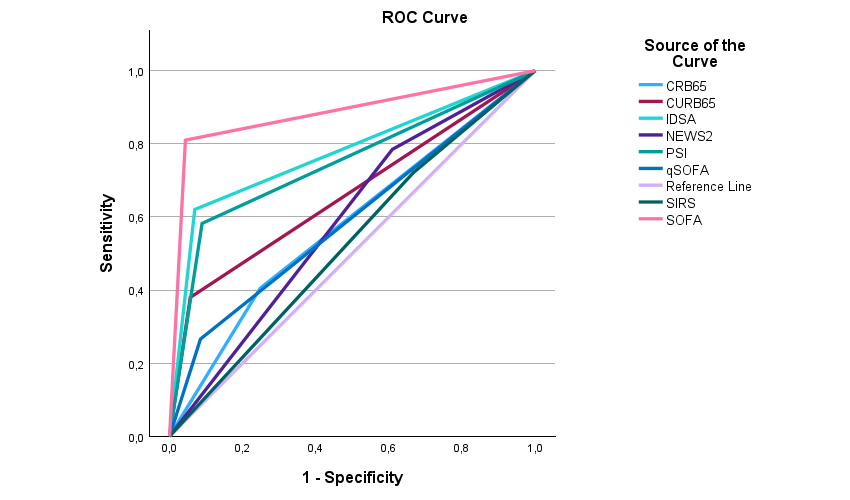


| **Area Under the ROC Curve** | | | | | | |
| --- | --- | --- | --- | --- | --- | --- |
| Test Result Variable(s) | Area | Std. Error^a^ | Asymptotic Sig.^b^ | Asymptotic 95% Confidence Interval | |  |
|  |  |  |  | Lower Bound | Upper Bound |  |
| SOFA | ,883 | ,026 | ,000 | ,832 | ,935 |  |
| IDSA | ,776 | ,034 | ,000 | ,710 | ,842 |  |
| PSI | ,747 | ,034 | ,000 | ,679 | ,814 |  |
| CURB65 | ,662 | ,037 | ,000 | ,589 | ,734 |  |
| NEWS2 | ,588 | ,031 | ,005 | ,526 | ,649 |  |
| qSOFA | ,591 | ,037 | ,013 | ,519 | ,662 |  |
| CRB65 | ,579 | ,035 | ,025 | ,510 | ,647 |  |
| SIRS | ,527 | ,033 | ,416 | ,462 | ,592 |  |
| The test result variable(s): SOFA, IDSA, PSI, CURB65, NEWS2, qSOFA, CRB65, SIRS has at least one tie between the positive actual state group and the negative actual state group. Statistics may be biased. | | | | | | |
| a. Under the nonparametric assumption | | | | | | |
| b. Null hypothesis: true area = 0.5 | | | | | | |

# Scoring system outlining

## Quick SOFA


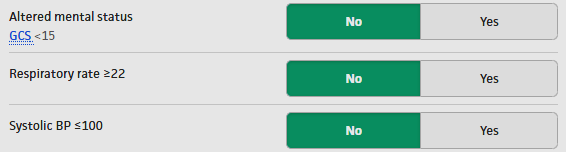


## SIRS


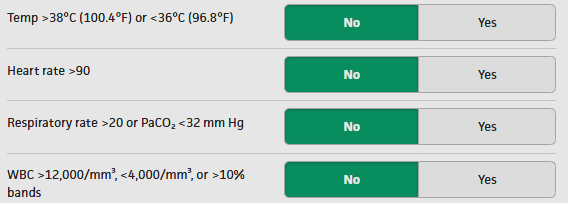


## NEWS2


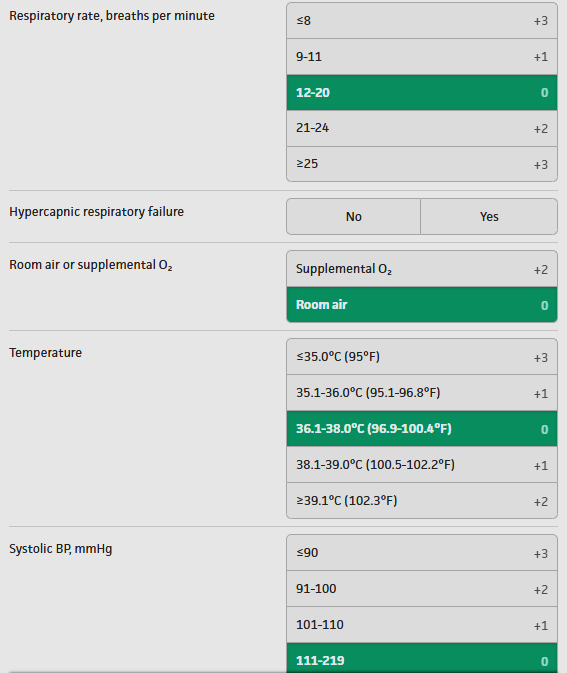


## CURB65


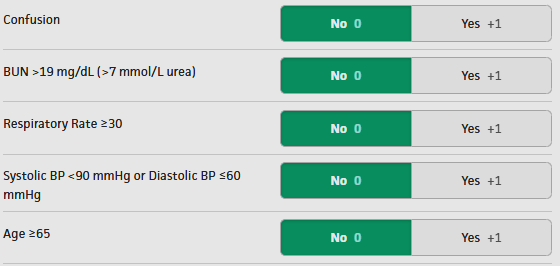


## CRB65


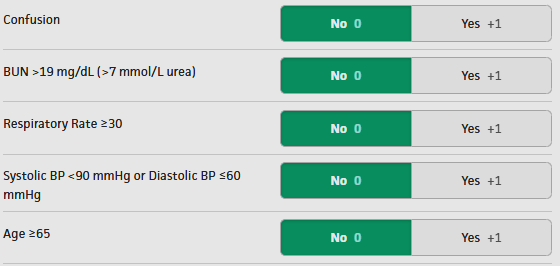


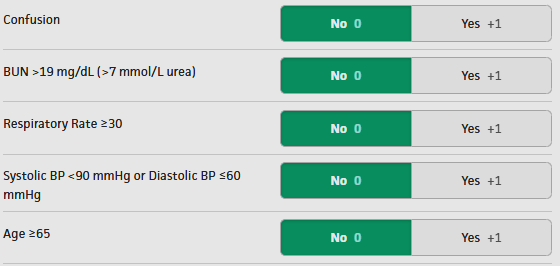


## IDSA/ATS


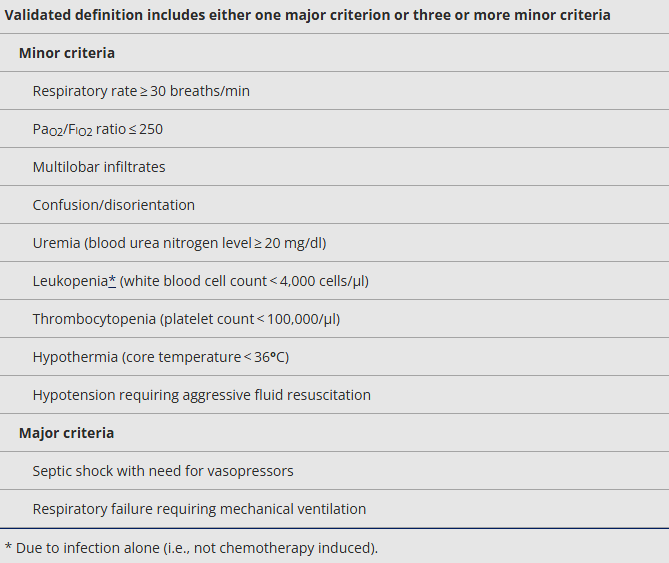


## PSI


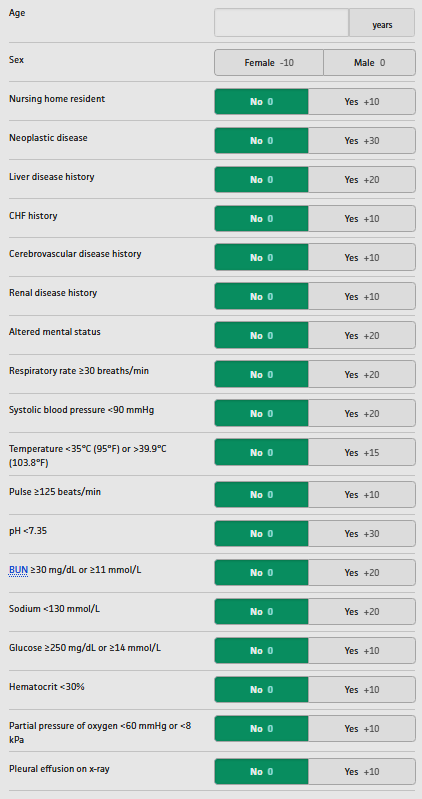


## SOFA


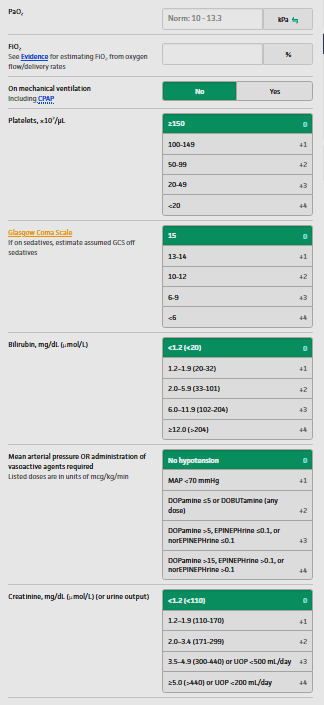

Supplement: Supplementary file 1 — Supplementary Material 1 [file 12890_2025_3550_MOESM1_ESM.docx]
